# Liver transplantation in metastatic colorectal cancer: are we ready for it?

**DOI:** 10.1038/s41416-023-02213-1

**Published:** 2023-03-06

**Authors:** Javier Ros, Francesc Salva, Cristina Dopazo, Daniel López, Nadia Saoudi, Iosune Baraibar, Ramon Charco, Josep Tabernero, Elena Elez

**Affiliations:** 1 Department of Medical Oncology, Vall d’Hebron Institute of Oncology (VHIO), 08035 Barcelona, Spain; 2 Medical Oncology, Department of Precision Medicine, Università degli Studi della Campania Luigi Vanvitelli, 80131 Naples, Italy; 3 Department of HPB Surgery and Transplants, Vall d’Hebron Hospital Universitari, Vall d’Hebron Institut de Recerca (VHIR), Vall d’Hebron Barcelona Hospital Campus, Universitat Autónoma de Barcelona, Barcelona, Spain

**Keywords:** Colorectal cancer, Surgical oncology

## Abstract

Colorectal cancer (CRC) is a prevalent disease worldwide, with more than 50% of patients developing metastases to the liver. Five-year overall survival remains modest among patients with metastatic CRC (mCRC) treated with conventional therapies however, liver transplantation in a highly selected population can improve clinical outcomes with an impressive 5-year overall survival of 83%. Despite liver transplantation appearing to be a promising therapeutical option for well-selected patients with mCRC with the liver-limited disease, these data come from small monocentric trials which included a heterogeneous population. Currently, several clinical trials are evaluating liver transplantation in this scenario, aiming for a more accurate patient selection by integrating liquid biopsy, tissue profiling, and nuclear medicine to the already known clinical biomarkers that eventually may lead to a survival improvement. In this paper, the clinical outcomes and inclusion criteria from the most relevant clinical trials and clinical series involving liver transplantation in patients with liver-limited disease colorectal cancer are reviewed as well as the trials currently recruiting.

## Introduction

Colorectal cancer (CRC) is the third most common cancer globally and the second most common cause of cancer-related deaths [1]. At diagnosis, 20–25% of patients have Stage IV disease, with 15–25% of cases presenting synchronous liver metastases [2]. Importantly, in 70–80% of these cases, metastases are confined to the liver [3]. The development of liver metastases in CRC has a strong impact on overall survival (OS), with median 5-year OS less than 14% with palliative chemotherapy [4–6]. In the management of liver-limited metastatic CRC (mCRC), locoregional treatment (surgical resection, thermal ablation, intraarterial chemotherapy, chemo- or radioembolization, radiotherapy, etc.) alone or in combination with systemic chemotherapy are considered appropriate alternative approaches [7]. Surgical treatment of liver metastases can prolong survival and even result in cure, achieving 5‐ and 10‐year survival rates of 42% and 25%, respectively [6, 8]. Unfortunately, liver resection is not always feasible, and the definition of non-resectable liver metastases is still not universally well-established, further complicating this issue. Furthermore, relapse rates after liver resection are high; 3-year relapse rates are 60–70%, with the liver being the most common site of recurrence [9–13]. Unlike the case of liver metastases arising from hepatocellular carcinoma (HCC), hilar cholangiocarcinoma, and low-grade neuroendocrine malignant disease where liver transplantation (LT) has demonstrated improved outcomes [14, 15], LT for CRC liver metastases is still under debate. Early attempts were performed in the 80 s with poor outcomes with a median 5-year OS of 18%; this strategy being abandoned considering the perioperative mortality rates of up to 30%, the high tumour relapse rates, poor survival, and shortage of donors [16, 17]. Fortunately, surgical techniques and survival after LT have improved over the last 30 years, while patient selection and immunosuppressive agents also contribute [18]. Over the last 20 years, few trials have evaluated the impact of LT among patients with mCRC. However, more recent trials evaluating the role of LT in this population have shown improved OS, likely related to more demanding inclusion criteria and a better understanding of predictive factors associated with recurrence and OS. Here, we review data from published clinical trials in this setting. Table 1 summarises patient characteristics and clinical outcomes of the reported clinical trials that are described in the following sections.

**Table 1:** Patient characteristics and clinical outcomes of clinical trials and most relevant cases series.

	TRIAL % (*n*)			
	SECA-I [19]	TOSO et al. [21]	SECA-II [22]	SECA-II Arm D [23]
Number of patients	21	12	15	10
Gender				
Male	62% (13)	50% (6)	53.3% (8)	70% (7)
Female	38% (8)	50% (6)	46.6% (7)	30% (3)
Age in years, median (range)	56 (45–65)	56 (38–73)	59.4 (34–71)	54 (30–70)
Primary tumour side				
Left	NS	NS	86.6% (13)	40% (4)
Right	NS	NS	13.3% (2)	60% (6)
TNM				
T1	0	0%	6.7% (1)	0%
T2	9.5% (2)	8.3% (1)	13.3% (2)	20% (2)
T3	76.1% (16)	66.6% (8)	73.3% (11)	70% (7)
T4	14.2% (3)	16.6% (2)	6.6% (1)	10% (1)
N0	33.3 % (7)	41.6% (5)	53.3% (8)	20% (2)
N1	33.3% (7)	41.6% (5)	40% (6)	0%
N2	33.3% (7)	16.6% (2)	6.6% (1)	80% (8)
N prior chemotherapy lines				
1	43% (9)	NS	46.7% (7)	100% (10)
2	38% (8)		40% (6)	100% (10)
3	19% (4)		13.3 (2)	30% (3)
Study type	Prospective	Retrospective	Prospective	Prospective
*RAS* mutation	NS	NS	6.7% (1)	30% (3)
*BRAF* mutation	NS	NS	0%	20% (2)
Time from primary surgery to LT in months, median (range)	36 (16–59)	41 (12–97)	22.6 (13–112)	16.5 (4–173)
Median N liver metastasis (range)	8 (4–40)	9	12 (3–100)	20 (1–45)
Diameter of largest liver metastases in cm, median (range)	4.5 (2.8–13)	2.1 (1–6)	2.4 (3–4.7)	5.9 (1.5–9.4)
Fong criteria (FCRS)				
0–2	24% (5)	Median 2.7 (range 1–4)	Median 2 (range 1–3)	Median 3 (range 2–5)
3–5	76.19% (16)			
Oslo criteria (median)	1 (0–4)	1 (range 0–2)	1 (range 0–1)	1 (range 1–4)
CEA prior to LT in ng/mL, median (range)	15 (1–2002)	16.9	2 (1–30)	4 (2–4346)
Follow-up in months, median (range)	27 (8–60)	26 (0–108)	36 (5–60)	23 (10–26)
Disease-free survival				
1 year	35%	56%	53%	30%
3-year	0%	38%	35%	NR
5-year	NA	38%	NR	NR
Overall survival				
1 year	95%	83%	80%	70%
3-year	68%	62%	40%	NR
5-year	60%	50%	13%	NR
Time to recurrence in months, median (range)	8 (2–24)	11.8 (0–108)	13.7 (NA)	4 (3–16)
Recurrence				
Lung alone	33.3% (7)	41.6% (5)	40% (6)	50% (5)
Liver	33.3% (7)	25% (3)	6.6% (1)	10% (1)
Other	52.3% (11)	8.3% (1)	13.3% (2)	20% (2)
Resection after relapse	47.6% (10)	0	53.3% (8)	0

*FCRS* Fong Clinical Risk Score, *LT* transplantation, *NS* not specified, *NR* not reached, *NA* not available.

## Development of liver transplantation in mCRC with liver metastases

### The first steps towards liver transplantation in mCRC

**Fig. 1:**
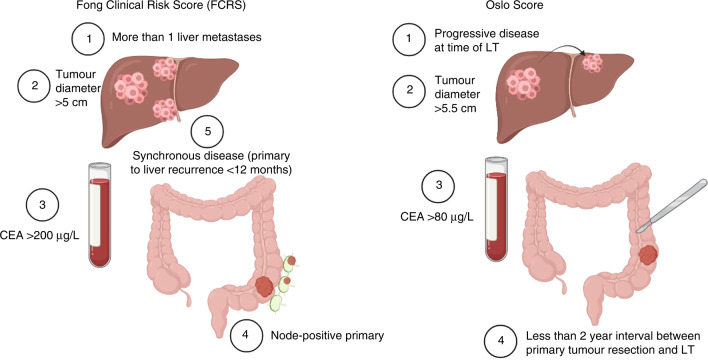
Comparison of Oslo Score, and Fong Clinical Risk Score criteria.

Taking advantage of the advances in surgical techniques and immunosuppressive drugs that led to a meaningful improvement in patients undergoing LT, a Norwegian group designed the first clinical trial to evaluate the impact of LT in patients with mCRC with liver-limited disease. The SECA-I study (NCT01311453) included 21 patients who underwent LT [19]. The main inclusion criteria in this trial were to have had radical excision of the primary tumour, good performance status (Eastern Cooperative Oncology Group [ECOG] 0–1), and a minimum of 6 weeks of chemotherapy (without a requirement in terms of response). The absence of extrahepatic disease was confirmed with positron emission tomographic CT scan (PET-CT). Of note, there was no information regarding the molecular status of any of the included patients. The Fong Clinical Risk Score (FCRS) was determined for each patient to give an estimation of prognosis. This score integrates nodal status of the primary tumour, and a disease-free interval between the primary tumour and liver metastases of <12 months, number of metastases >1, preoperative carcinoembryonic antigen (CEA) level >200 ng/mL, and size of the largest tumour >5 cm, to predict OS in patients with liver metastases (Fig. 1). The score ranges from 0 to 5 points, with higher scores reflecting worse survival (median OS was 74, 51, 47, 33, 20 and 22 months for patients with scores of 0, 1, 2, 3, 4 and 5, respectively) [20].

In SECA-I, at baseline, 57% of the patients had received ≥2 chemotherapy lines, 38% had >10 liver metastases, and 76% had an FCRS of 3–5 [19]. After a median follow-up of 27 months, 1-year and 5-year OS rates were 95% and 60% respectively, and the disease-free survival (DFS) rate at 1 year was 35%. The major impact of the SECA trial was not only the impressive 5-year OS rate but also the identification of several factors influencing outcomes. Among known prognostic factors associated with CRC liver metastases, four were found to be significantly associated with survival: lesion diameter (largest tumour diameter ≥5.5 cm), <2 years since primary tumour resection, elevated CEA levels (>80 ng/mL), and progressive disease at the time of LT. Based on the sum of these risk factors, patients were categorised in three subgroups—0–1 factors, 2–3 factors and 4 factors—with different survival outcomes. This score is known as the Oslo prognostic score (Fig. 1). Interestingly, this study also showed that patients with high PET-CT uptake in liver metastases before LT had worse OS.

Regarding disease recurrence, the median time to recurrence was 8.0 months. The most frequent involved organ was the lung (80%), followed by the transplanted liver (33%). Of note, a high proportion (38%) of patients with recurrence after LT were eligible for surgery or radiofrequency treatment. None of the patients died of surgical complications. Despite the impressive 60% 5-year OS, some limitations of the study should be highlighted: the sample size was small, and there was a high recurrence rate and a short DFS. However, considering that 5-year OS is less than 10% after starting first-line chemotherapy, these results showed an encouraging impact in terms of survival. Furthermore, it should also be considered that this patient cohort was heavily pretreated, had generally high FCRS, and a high number of metastases.

### Subsequent studies tailored prognostic factors and inclusion criteria

Recently, a retrospective multicentre study included 12 patients who underwent LT. In this small cohort, patients had received a median of two prior chemotherapy lines and all patients had responded to the treatment [21]. LT was performed a median of 41 months (range, 12–97 months) after the primary tumour resection. OS at 1, 3 and 5 years was 83%, 62% and 50%, respectively, after a median follow-up of 26 months (range, 0–108 months). The lungs were the most frequent site of relapse (42%), followed by the liver (25%). DFS at 1, 3 and 5 years was 56%, 38%, and 38%, respectively. The time between primary tumour resection and LT was the most relevant prognostic factor followed by pre-LT CEA levels, as established by the Oslo criteria. This study moved a step forward over the SECA trial, demonstrating that longer DFS can also be achieved. It highlights the importance of CEA levels and the time between primary tumour resection and transplantation as well as the impact of undergoing LT in the context of chemotherapy response. However, this study did not include any information regarding the tumour molecular biology.

The SECA-II study (NCT01479608), also performed by the Norwegian group, was a prospective trial including 15 mCRC patients with non-resectable liver-limited disease, and had more restrictive selection criteria than SECA-I [22]. Patients had to have had a PET-CT scan, have an ECOG performance status 0–1, have received first-line treatment, and at least a 10% response measured with Response Evaluation Criteria In Solid Tumours (RECIST) after chemotherapy. Baseline characteristics of the patients included 47% (7/15) receiving first-line chemotherapy, and one patient had disease harbouring a *KRAS* mutation. The median number of lesions at the time of LT was 5 (range, 1–5) and the median FCRS was 2 (range, 1–3). After a median follow-up of 36 months, OS rates at 1, 3 and 5 years was 100%, 83% and 83%, respectively. DFS rates at 1, 3 and 5 years were 53%, 44% and 35% respectively, with a median of 13.7 months (range, 5–60 months). As expected, patients with FCRS 0–2 had longer median DFS compared with patients with a FCRS of 3–4 (11.8 months vs not reached). Six of the eight patients with relapse after LT developed pulmonary metastases as the first or only site of metastatic disease, with five undergoing surgical resections. It must be highlighted that a total of 11 patients had no evidence of disease at the end of follow-up. Compared with the SECA-I trial, none of the SECA-II patients had progressive disease on chemotherapy or CEA levels above 80 μg/L at the time of LT. Furthermore, patients enrolled in SECA-I presented a high Oslo Score at the time of LT (median 2 vs 1), a higher median number of liver lesions (8 vs 5), larger lesions (median 45 vs 24 mm), higher CEA levels (median 15 vs 2 μg/L), and high SUVmax in the PET-CT (median 9 vs 5.9), suggesting that more accurate patient selection may lead to improved survival.

The Norwegian group recently published the results of the arm D from the SECA-II trial (NCT01479608) which for multiple reasons did not meet the strict criteria for the arms A, B and C of the SECA-II study, as it applied extended donor criteria considered unacceptable for patients on the regular waiting list [23]. The group enrolled 10 patients with non-resectable CRC liver metastases. All patients had received two or three lines of chemotherapy before LT and two had progressive disease on the last line of chemotherapy before LT. Three patients had *KRAS* mutations (type not specified) and two presented tumours harbouring *BRAF* mutations (type not specified). After a median follow-up of 23 months, 60% (6/10) of the patients had pulmonary relapse as the first site of relapse. Median DFS and OS were 4 months and 18 months respectively. Interestingly, this is the unique trial to report survival in terms of tumour side; patients with a right-sided primary tumour had a median DFS of 3 months, and all patients with right-sided tumour relapsed within 16 months of LT, whereas the four patients with a left-sided primary tumour had a median DFS of 10 months and two patients had not relapsed 23 and 26 months after LT. Regarding the *BRAF* mutant patients, one patient had an OS of 6 months and the other was still alive 26 months after LT with no evidence of relapse. The eight patients with *BRAF* wild type had a median OS of 18 months. Despite a clearly different patient profile compared with patients enrolled in the SECA-I and SECA-II trials, this trial population confirmed the already known prognostic factors.

Bearing this in mind along with the results of these trials, further development of selection criteria for LT in CRC patients with unresectable liver-limited disease may allow selection of patients who will achieve 5-year OS similar to that of patients who currently receive LT as standard of care. As a pilot exploratory study, SECA-I included a heterogenous population regarding prognostic factors such as number and size of lesions, CEA level, use and response to chemotherapy, and time from cancer diagnosis to LT. The SECA-II had stricter inclusion criteria with an interval from diagnosis to LT of at least 1 year and required at least 10% response to chemotherapy, thus gathering a more selective group of patients with a better prognostic. As reported in the literature, the lungs are the most frequent site of relapse and half of these patients can be treated with curative intent [24]. Furthermore, PET-CT is required in all trials, not only to rule out extrahepatic metastases but also because PET-CT uptake has been demonstrated to have prognostic value [25]. Indeed, SUV-based metabolic parameters using 18F-FDG PET-CT predicted outcomes in patients with mCRC and liver-limited disease who previously underwent resection of their liver metastases. In addition, SUVmean, SUVmax, total lesion glycolysis(TLG) metabolic tumour volume and haemoglobin level have all been shown to be associated with longer RFS and PFS in different studies [25, 26]. In the SECA trial, 18F-FDG PET-CT previous to the liver transplant demonstrated that total metabolic tumour volume (MTV) and TLG were significantly correlated to improved OS at 3 and 5 years [27]. Thus, liver transplantation can achieve long survival in among well-selected patients with mCRC.

### Choice of inclusion criteria and the International Consensus recommendation for LT

In 2021, an international consensus guideline for LT for non-resectable CRC liver metastases was published [28]. The guideline aims to improve patient selection to achieve optimal clinical outcomes by excluding patients with high-risk factors for relapse. In terms of clinicopathological and radiological criteria, the guidelines state that the primary tumour must be removed with clear resection margins, and undifferentiated adenocarcinomas and signet ring-cell carcinomas should be excluded. Nodal disease of N2 stage for the primary tumour should be excluded. PET-CT is recommended for all patients to rule out other non-liver metastases, and metabolic tumours with a volume >70 cm^3^ and total lesion glycolysis of >260 g should be excluded. There are no exclusion recommendations concerning sidedness or the number and size of metastatic lesions. Regarding molecular biomarkers, while patients with primary tumours harbouring *BRAF* mutation should not be considered for LT, there is no specific contraindication for *RAS* mutations, although these mutations are associated with a slightly worse prognosis compared with wild-type tumours. Interestingly, because of the favourable results with immunotherapy in patients with microsatellite instability (MSI) or mismatch repair deficient mCRC, the role of LT in these patients should be carefully discussed. It must be highlighted that given the meaningful impact of liquid biopsy in the adjuvant setting as a paramount prognostic factor, extended molecular profiling is strongly recommended in the clinical research setting. Furthermore, patients should have received at least one line of fluorouracil-based, oxaliplatin-based, or irinotecan-based therapy, with response for at least 6 months, and matched therapy might be considered in patients with specific actionable mutations. Radiological or biochemical progression remains a contraindication for LT. Radiological assessment should be based on RECIST. Biochemical response evaluation is based on plasmatic CEA levels. CEA > 80 μg/L with an increasing trend is a contraindication whereas CEA > 80 μg/L with a decreasing trend is only a relative contraindication.

Another point of interest is the choice of graft selection and allocation. This will depend on organ availability and waiting list mortality, following as far as possible, the ethical principle of usefulness. Most countries follow the ‘sickest-first’ approach based on the model for end-stage liver disease (MELD) score, and inclusion of patients with malignant indications such as hepatocellular carcinoma or perihilar cholangiocarcinoma on the LT waiting list, is allocated with MELD concession points to increase their waiting list priority. In countries with greater availability of deceased donors, the same practice could be applied for patients with non-resectable CRC liver metastases. Based on the Norwegian experience, this would only increase the annual LT volume by 1–2% [29]. In countries with a shortage of donor organs, living-donor LT could be offered for this indication in high-experience centres, where even the use of extended criteria donor grafts is recommended. Novel surgical techniques to expand the donor pool should also be encouraged, as well as the use of ex situ machine perfusion technologies to recover non-transplantable livers, and resection and partial liver segment transplantation (deceased or living donor) using the delayed total hepatectomy (RAPID) technique [30]. Liver grafts have lower alloreactivity compared to other organs with a very low rate of graft loss (<5% at 1 year) because of acute rejection [31]. Thus, calcineurin inhibitor minimisation strategies using low doses of tacrolimus for long-term maintenance with the addition of an mTOR inhibitor is highly recommended. Finally, if liver transplantation is considered, quality of life must be also taken into consideration. The quality of life of the liver transplanted cohort from the SECA trial (using the QLQ-C30 questionnaire) was compared to data obtained from a cohort of patients with metastatic colorectal cancer receiving first-line chemotherapy. Patients in both cohorts reported similar baseline QoL score, although patients from the SECA trial had previously received chemotherapy. Despite a relapse, in most of the liver transplanted patients, the Global Health Score remained good [32]. Thus, patients who received liver transplantation had a good long-term quality of life, and patients with high symptom score before transplantation had worse overall survival conversely to patients with low symptom score [33].

## Future trials

At the time of writing there were 15 ongoing clinical studies evaluating the role of LT in patients with liver-limited mCRC, which are summarised in Table 2. These trials evaluate several aspects of this therapeutic strategy, including LT vs. chemotherapy, liver resection vs. LT for resectable CRC liver metastasis, and assessment of new means to expand the donor pool of liver grafts (living donor, extended, or partial grafts). With the aim to optimise patient selection criteria for LT, most of the ongoing studies include molecular tumour markers in their inclusion criteria, and exclude patients with *BRAF* mutant tumours or MSI.

**Table 2:** Ongoing clinical trials evaluating liver transplantation as a therapeutic strategy in mCRC.

Trial ID/name	Country	Study design	Donor graft	Brief description	Main inclusion criteria	Primary endpoint	Enrolment goal/Status
NCT01479608 (SECA-II)	Norway	Phase II	Deceased donors	Evaluate LT vs surgical resection in terms of significant life extension and health-related quality of life	*Experimental A:* LT or liver resection by 1:1 randomisation (open label) for patients with ≥6 resectable lesions *Experimental B:* LT for non-resectable patients with metachronous disease diagnosed >12 months from CRC resection and pN0 *Experimental C:* LT for non-resectable patients with synchronous disease. Time from primary CRC resection to LT ≥2 years *Experimental D:* LT for non-resectable patients with synchronous disease; resectable pulmonary disease permitted	10-year OS	25/Recruiting
NCT03494946 (SECA-III)	Norway	Randomised controlled open-label	Deceased donors	Compare LT vs chemotherapy alone	Exclusion of patients with three negative prognostic factors at time of randomisation (CEA > 80 μ/L, < 2 years since diagnosis, diameter of largest liver lesion >5.5 cm) 1–3 resectable lung lesions all <15 mm	2-year OS	30/Recruiting
NCT02215889 (RAPID)	Norway	Phase I/II	Left lateral segment	Evaluate two-stage hepatectomy combined with left lateral liver graft	Time from primary CRC resection to LT ≥12 months Systemic chemotherapy for ≥8 weeks 1–3 resectable lung lesions all <15 mm	% transplanted patients with second-stage hepatectomy <4 weeks after left lateral segment transplantation	20/Recruiting
NCT02597348 (TRANSMET)	France	Randomised parallel open-label	Deceased donors	Compare LT vs chemotherapy alone	High-standard resection of primary CRC without local recurrence Systemic chemotherapy for ≥3 months and ≤2 lines CEA < 80 μ/L or ≥50% decrease of highest serum CEA *BRAF* wild type	5-year OS	90/Active, not recruiting
NCT02864485	Canada	Pilot	Living donor	Evaluate living-donor LT for non-resectable CRC metastases	Living donor available Primary CRC ≤T4a Time from primary CRC resection to LT ≥6 months Systemic chemotherapy for ≥3 months *BRAF* wild type	5-year OS 5-year DFS	20/Recruiting
NCT03488953 (LIVER-T(W)O-HEAL)	Germany	Pilot	Left lateral segment Living donor	Evaluate two-stage hepatectomy combined with left lateral liver graft from living donor	Systemic chemotherapy for ≥8 weeks Resectable lung lesions permitted	3-year OS after second hepatectomy	40/Recruiting
NCT04161092 (SOULMATE)	Sweden	Randomised open-label parallel	Deceased donors	Evaluate LT with liver grafts from extended criteria donors not used for approved indications for non-resectable isolated liver metastases from CRC, vs best alternative care	Time from primary CRC diagnosis to LT ≥12 months Systemic chemotherapy for ≥4 weeks *BRAF* wild type and MSS	5-year OS	45/Recruiting
NCT03803436 (COLT)	Italy	Non-randomised open-label parallel	Deceased donors	Compare survival in the COLT trial with COLT-eligible patients in the GONO Group TRIPLETE trial	Primary tumour pT1-3, pN0 or pN1 Systemic chemotherapy for ≥4 weeks and ≤2 lines CEA < 50 μ/L *RAS* and *BRAF* wild type and MSS	5-year OS	22/Recruiting
NCT04870879 (MELODIC)	Italy	Non-randomised open-label parallel	Deceased donors	Compare survival in the MELODIC trial with a matched cohort treated with chemotherapy	Time from primary CRC resection to LT ≥10 months Systemic chemotherapy for ≥3 months Liver metastases ≤10 cm at diagnosis CEA < 100 μ/L *BRAF* wild type	3-year OS 3-year DFS	18/Recruiting
NCT04865471(RAPID-PADOVA)	Italy	Pilot	Left lateral segment	Evaluate two-stage hepatectomy in combination with left lateral liver graft	Time from primary CRC resection to LT ≥6 months Systemic chemotherapy for ≥3 months Patients may have 1–3 resectable lung lesions all <15 mm *BRAF* wild type	% of transplanted patients receiving second-stage hepatectomy within 4 weeks of segment 2/3 transplantation	18/Recruiting
NCT04616495 (TRANSMETIR)	Spain	Observational (patient registry)	Deceased donors	Evaluate LT for non-resectable CRC metastases well controlled by chemotherapy prior to transplantation	Time from primary CRC diagnosis to LT ≥12 months Systemic chemotherapy ≤2 lines CEA < 80 μ/L Liver metastases ≤5 cm (in last imaging) *BRAF* wild type	5-year OS	30/Recruiting
NCT04898504 (EXCALIBUR 1 + 2)	Norway	3-armed randomised controlled open label	Deceased donors	Evaluate second-line chemotherapy + HAI-floxuridine or LT vs second-line chemotherapy alone vs HAI + LT + second line chemotherapy alone	>6 liver metastases that have progressed (or insufficient response on first-line chemotherapy, including toxicity) Planned for second-line chemotherapy Any history of confirmed extrahepatic metastases or local relapse must be successfully treated >2 years ago without new relapse	2-year OS	45/Recruiting
NCT04742621	US	Observational (patient registry)	Deceased donors	Evaluate LT for non-resectable CRC metastases well controlled by chemotherapy prior to transplantation	Time from primary CRC resection to LT ≥6 months and 1 year since diagnosis Systemic chemotherapy for ≥6 months CEA < 200 μ/L *BRAF* wild type and MSS	LT registry of patients with liver-limited mCRC at Weill Cornell Medical College/New York-Presbyterian Hospital	20/Recruiting
NCT04874259	South- Corea	Pilot study	Living donor	Evaluate living-donor LT for non-resectable CRC metastases	Documented untreated CRC liver metastases Liver-limited	1-year OS	20/Not yet recruiting
NCT05398380 (METLIVER)	Spain	Phase II	Deceased donors	Evaluate LT for non-resectable CRC metastases, to expand genetic studies and impact of circulating tumour DNA	Time from primary CRC resection to LT ≥12 months Systemic chemotherapy for ≥3 months and ≤ 2 lines Primary CRC ≤T3N1 or T4N0 CEA < 80 μ/L Liver metastases ≤5.5 cm (in last imaging) *BRAF* wild type and MSS	5-year OS	35/Recruiting

*CEA* carcinoembryonic antigen, *CRC* colorectal cancer, *DFS* disease-free survival, *HAI* hepatic artery infusion, *LT* liver transplantation, *MSS* microsatellite stability, *OS* overall survival.

## Discussion

Liver metastases remain a major cause of death among patients with liver-limited CRC despite improvements resulting in more appropriate systemic treatments and less restrictive surgical approaches. Recent advances have demonstrated that LT can improve 5-year OS, reaching 80% in a highly selected population. While the Oslo and the Fong scores can identify patients with poor prognosis, other risk factors should also be taken into account [29, 34]. Conversely, in the refractory setting, both TAS-102 and regorafenib have demonstrated modest improvement in OS compared to the placebo (1.8 and 2.4 months, respectively) [35, 36]. Other strategies such as the combination of chemotherapy and hepatic artery infusion demonstrated a 5-years DFS of 10% and a potential cure rate of 9%. These results seem promising, but inferior compared to the SECA studies [37] and it should be considered that the inclusion criteria were not exactly the same as for liver transplantation. Updated results from the SECA trial showed a 10-years OS of 26.1%, and 4 patients remained free of relapse after a median of 102 months. After this longer follow-up, 10-year OS was 50% and 33% for those patients with Oslo score of 0–1 and Oslo score 2 respectively. All patients with Oslo score 3–4 were deceased 86 months after liver transplant [38]. Clinical evidence comes principally from three clinical trials and one retrospective cohort, all with modest sample sizes and heterogeneous populations, and without taking into consideration meaningful prognostic factors that have been identified over the last 10 years such as tumour sidedness, patient performance status or molecular biology including *RAS/BRAF* mutations [7, 39–41]. Results from clinical trials evaluating outcomes after LT in mCRC patients have shown that the pattern of recurrence and their natural history after LT differs from that of recurrence after liver resection for liver-limited disease [42]. After hepatectomy, most recurrences were in the remnant liver, with only 26% of patients developing isolated lung metastases [43] whereas after LT, recurrence is mostly pulmonary, isolated, and amenable to curative surgery [44, 45]. Furthermore, pulmonary metastases after LT were found to be slow growing and, as previously mentioned, in many cases accessible to surgical therapy. Regarding lung metastasis, findings in the SECA group were compared with patients in a control group with non-transplanted, resectable, lung-limited rectal cancer. It was demonstrated that LT immunosuppression did not have an impact not only in terms of median doubling time based on tumour diameter and volume but also in the metastasis distribution and disease-free survival [46]. These results suggest that immunosuppression after LT does not accelerate the growth of pulmonary metastases and that pulmonary recurrence does not necessarily preclude long-term survival. This suggests that LT has a favourable recurrence profile compared to recurrence after liver resection [47]. That being said, and considering that most of the patients will have cancer recurrence after liver transplantation, DFS is of limited value as a parameter of treatment efficacy and it may be an inadequate surrogate point for overall survival in this scenario [48].

The 5-year OS expected for those patients with HCC within Milan criteria is 70%. However, since liver transplant has demonstrated a very good long-term OS in those patients with HCC outside Milan criteria compared to locoregional therapies, a 5-year OS around 60% is considered optimal [49] as it is for perihilar cholangiocarcinoma [50] and for CRC liver metastases [29]. However, it will depend on the organ availability of each country to accept these new indications. Indeed, shortage of organs is one major concern related to the use of organs for transplantations among patients with cancer. The policy on liver transplantation depends on each country’s regulations. Some protocols prioritise the living donor, and most of the countries include cancer patients on the same waiting list which include patients with non-cancer indications with the same possibilities to receive a brain donor, deceased donor or after ex situ machine perfusion. Notwithstanding the aforementioned facts, liver transplantation for patients with CRC liver-limited disease seems to be an option only for a small minority of carefully selected patients. In the SECA trial, only 1–2% of the evaluated patients were finally accepted for transplantation, which represents 0.24–0.51 patients per million people per year [34].

Clinicians are thus faced with a dilemma that on the one hand, the more restrictive the patient selection criteria, the better the outcome whereas, on the other hand, if criteria are more permissive, disease will certainly recur but with a more indolent profile despite not achieving cure. This is indeed a challenging decision. Management of the LT waiting list and defining fair organ prioritisation is extremely complex. A key issue is therefore access to liver grafts, which varies between countries. In countries with shorter waiting lists, more palliative transplantation could be considered accepting higher recurrence rates, in an attempt to minimise the impact on other patients on the waiting list [51]. Furthermore, to increase the number of available liver grafts, living-donor LT has also been explored. In a recent cohort of ten patients with non-resectable liver metastases from CRC, living-donor LT was performed. Two of the ten patients had a right-sided primary tumour. *KRAS* mutations were detected in three patients and one patient presented a *BRAF D594G* mutation. The median Oslo score was 1.5 (range, 0–2). The recurrence-free and OS rates at 1.5 years were 62% and 100%, respectively [52], which are consistent with those reported in the SECA-II study. Considering the results and the fast-moving pace of LT management in CRC, it would be advisable to start with more restrictive inclusion criteria before being more inclusive. Based on current clinical evidence, molecular data and liquid biopsy (for example, plasmatic *RAS* or *BRAF* mutant allele fraction) [53, 54] should be strongly considered as stratification tools for better patient selection, however, further and prospective data in the specific scenario of liver transplantation is needed. Over the last decade, clinical care of mCRC has been improved by the identification of several genomic alterations with prognostic and predictive impact [55]. The encorafenib-cetuximab combination for *BRAF-V600E* mutant mCRC is a clear example of how targeted agents reshape disease evolution [56, 57]. In the case of patients with mCRC and mismatch repair deficiency or MSI, responses to the anti-PD1 pembrolizumab have had a deep impact on clinical outcomes [58]. More recently, drugs targeting *KRAS G12C* or *HER2* have also improved survival in patients with metastatic disease [59–61]. Despite these clinical improvements and advances in personalised medicine, most patients will ultimately relapse. This leaves a window of opportunity to consider LT as a treatment strategy for mCRC patients.

Importantly, a major concern in relation to LT in mCRC is the exclusion of patients with solid-organ transplantation from clinical trials, reflecting the loss of a potential treatment opportunity. Few of the described trials reported *RAS* and *BRAF* mutations or sidedness. Considering the survival impact of targeted agents and the continuous development of novel biomarkers, it is fundamental to have complete molecular information to help clinicians establish an accurate prognosis and define the most appropriate treatment. Another meaningful prognostic factor is the sidedness of the primary tumour. The prognostic and predictive impact of the primary tumour side has been widely and clearly demonstrated [62–64]. Right-sided tumours are less responsive to anti-EGFR agents and are associated with worse prognosis compared with left-sided tumours. Of note, only one of the aforementioned clinical trials considered tumour location as a prognostic factor. This demonstrates that not only molecular features but also sidedness should be considered when implementing the decision about LT. Finally, in the last five years, liquid biopsy for detecting circulating tumour DNA (ctDNA) has proven to be a robust prognostic marker of relapse-free survival in localised CRC. Several trials have demonstrated that patients with a positive post-surgical liquid biopsy are at higher risk of relapse compared with patients with a negative liquid biopsy [65–67]. Indeed, the detection of post-surgical circulating tumour DNA was an independent negative prognostic marker and identifies those patients at high risk of relapse after liver metastases resection [68]. Future studies should include all these prognostic factors to tailor prognostic forecasts and treatments and take them all into consideration to the most appropriate treatment strategy. Another concern regarding the impact of LT in cancer patients is that immunosuppressive treatment to avoid allograft rejection could boost cancer evolution and decrease efficacy of some cancer treatments such as immune-checkpoint inhibitors. However, in the particular case of immune-checkpoint inhibitors in patients with solid-organ transplantation, it has been demonstrated that the rejection rate is relatively low and immunosuppression does not compromise clinical activity [69]. Furthermore, there is still little evidence about the use of chemotherapy after LT and potential interactions with immunosuppressive drugs. A report of three such patients with LT for mCRC described no unexpected toxicities [70]. A pooled analysis of 23 patients, from three different clinical trials, with colorectal cancer and non-resectable liver metastases who underwent liver transplant evaluated the toxicity of post-transplant chemotherapy: Overall, chemotherapy for mCRC was well-tolerated and there was no increased bone marrow toxicity after liver transplant, however, mucositis and diarrhoea were more frequent in post-liver transplant chemotherapy [71]. However larger and prospective studies are needed to confirm the safety and potential complications of chemotherapy following LT. What is clear is that narrowing the inclusion criteria leads to better patient selection and improved survival. However, based on the current data, the role of LT in CRC liver metastasis remains exploratory and should for now remain limited to the clinical trial setting.

## In summary

Five-year OS remains modest among mCRC patients treated with conventional therapies, however, LT in a highly selected population can improve clinical outcomes with an impressive 5-year OS of 83% in one study. Although these data come from small monocentric trials involving a heterogeneous population, LT appears to be a promising therapeutical option for well-selected patients with mCRC with the liver-limited disease. Genomic analysis including liquid biopsy, tissue profiling, and nuclear medicine are paramount tools to perform accurate patient selection and should be integrated in future clinical trials to identify those patients who will achieve the greatest benefit from LT. Furthermore, to obtain meaningful benefit, a collaborative effort including surgeons, medical oncologists, and translational researchers is needed. Considering the importance of establishing prognostic factors for LT in this population in the mCRC setting, for now, decisions are more important than incisions.

## Supplementary information

Author list change agreement

## Data Availability

Not applicable.
