# Correction To: Liver transplantation in metastatic colorectal cancer: are we ready for it?

**DOI:** 10.1038/s41416-023-02370-3

**Published:** 2023-07-24

**Authors:** Javier Ros, Francesc Salva, Cristina Dopazo, Daniel López, Nadia Saoudi, Iosune Baraibar, Ramon Charco, Josep Tabernero, Elena Elez

**Affiliations:** 1 Department of Medical Oncology, Vall d’Hebron Institute of Oncology (VHIO), 08035 Barcelona, Spain; 2 Medical Oncology, Department of Precision Medicine, Università degli Studi della Campania Luigi Vanvitelli, 80131 Naples, Italy; 3 Department of HPB Surgery and Transplants, Vall d’Hebron Hospital Universitari, Vall d’Hebron Institut de Recerca (VHIR), Vall d’Hebron Barcelona Hospital Campus, Universitat Autónoma de Barcelona, Barcelona, Spain

**Keywords:** Colorectal cancer, Surgical oncology

Correction to: *British Journal of Cancer* 10.1038/s41416-023-02213-1, published online 06 March 2023

Two typographical errors were present in Table 1 of the original article.

In the first column, the second range of Fong criteria (FCRS) should read 3–5; the original article read 2–5.

In the SECA-I column, the ‘Diameter of largest liver metastases in cm, median (range)’ should read 4.5 (2.8–13); the original article read 4.5 (2.8–1.3).

The original article has been corrected.

