# Supplementary material for: Liver transplantation in metastatic colorectal cancer: are we ready for it?
Source: Br J Cancer. 2023 Mar 6;128(10):1797–806. doi: 10.1038/s41416-023-02213-1 (PMC10147684; doi:10.1038/s41416-023-02213-1)
Supplement: Supplementary file 1 — Author list change agreement [file 41416_2023_2213_MOESM1_ESM.docx]

To whom may it concern,

We were requested by BJC to have all authors’ confirmation in order to include Dr Josep Tabernero as one of the authors of our review entitled  "Liver transplantation in metastatic colorectal cancer: Are we ready for it?" BJC-REV3341902R1.

All the authors have agreed with the inclusion of Prof Josep Tabernero. As requested, I attach all the e-mails confirming this fact:

**Iosune Baraibar Argota**

Para:

- FRANCISCO JAVIER ROS MONTAÑA;
- FRANCESC SALVA BALLABRERA;
- cdopazo

y 6 más

Jue 09/02/2023 16:36

Dear Javier,

I totally agree on including Prof Josep Tabernero as one of the authors.

Very best wishes,

Iosune

**Lopez Valbuena, Daniel Esteban <danielesteban.lopez@vallhebron.cat>**

Para:

- FRANCISCO JAVIER ROS MONTAÑA;
- FRANCESC SALVA BALLABRERA;
- cdopazo;
- NADIA SAOUDI GONZALEZ

y 4 más

Jue 09/02/2023 16:42

Dear Dr. Ros,

I completely agree with the inclusion of Dr. Josep Tabernero as one of the authors

Kind regards,

Daniel Esteban López Valbuena

**NADIA SAOUDI GONZALEZ**

Para:

- Iosune Baraibar Argota;
- FRANCISCO JAVIER ROS MONTAÑA;
- FRANCESC SALVA BALLABRERA;
- cdopazo

y 5 más

Jue 09/02/2023 16:42

Dear Javier,

I also fully agree on including Prof Josep Tabernero as one of the authors.

Best wishes,

Nadia

**FRANCESC SALVA BALLABRERA**

Para:

- NADIA SAOUDI GONZALEZ

CC:

- Iosune Baraibar Argota;
- FRANCISCO JAVIER ROS MONTAÑA;
- cdopazo

y 5 más

Jue 09/02/2023 16:43

Dear Javier

I also fully agree on including Prof Josep Tabernero as one of the authors.

Best wishes,

Francesc

**Dopazo Taboada, Cristina <cristina.dopazo@vallhebron.cat>**

Para:

- FRANCISCO JAVIER ROS MONTAÑA;
- FRANCESC SALVA BALLABRERA;
- cdopazo

y 6 más

Jue 09/02/2023 17:22

Dear Javier,

I  agree on including Prof Josep Tabernero as one of the authors.

Best wishes,

**Ramon Charco Torra <rcharco@gmail.com>**



Para:

- FRANCISCO JAVIER ROS MONTAÑA

Jue 09/02/2023 20:34

Dear Javier,

I agree to include Dr. Josep Tabernero as co-author.

Best regards,

**Elena Elez <meelez@vhio.net>**



Para:

- FRANCISCO JAVIER ROS MONTAÑA

Jue 09/02/2023 20:34

Dear Javier,

I agree to include Dr. Josep Tabernero as co-author.

E
